# Sequence-Based Mapping of the Polyploid Wheat Genome

**DOI:** 10.1534/g3.113.005819

**Published:** 2013-07-01

**Authors:** Cyrille Saintenac, Dayou Jiang, Shichen Wang, Eduard Akhunov

**Affiliations:** * Department of Plant Pathology, Kansas State University, Manhattan, Kansas 66502

**Keywords:** sequence-based genotyping, contig anchoring, gene mapping, reference map

## Abstract

The emergence of new sequencing technologies has provided fast and cost-efficient strategies for high-resolution mapping of complex genomes. Although these approaches hold great promise to accelerate genome analysis, their application in studying genetic variation in wheat has been hindered by the complexity of its polyploid genome. Here, we applied the next-generation sequencing of a wheat doubled-haploid mapping population for high-resolution gene mapping and tested its utility for ordering shotgun sequence contigs of a flow-sorted wheat chromosome. A bioinformatical pipeline was developed for reliable variant analysis of sequence data generated for polyploid wheat mapping populations. The results of variant mapping were consistent with the results obtained using the wheat 9000 SNP iSelect assay. A reference map of the wheat genome integrating 2740 gene-associated single-nucleotide polymorphisms from the wheat iSelect assay, 1351 diversity array technology, 118 simple sequence repeat/sequence-tagged sites, and 416,856 genotyping-by-sequencing markers was developed. By analyzing the sequenced megabase-size regions of the wheat genome we showed that mapped markers are located within 40−100 kb from genes providing a possibility for high-resolution mapping at the level of a single gene. In our population, gene loci controlling a seed color phenotype cosegregated with 2459 markers including one that was located within the red seed color gene. We demonstrate that the high-density reference map presented here is a useful resource for gene mapping and linking physical and genetic maps of the wheat genome.

Reference mapping populations are a very useful resource for genome analysis and understanding the genetic basis of phenotypic variation. These populations have been broadly used for genetic analyses of rice (http://www.gramene.org/species/oryza), maize (http://maizegdb.org/map.php), and *Arabidopsis* (Koornneef and Meinke 2010) genomes. The utility of reference populations significantly increases with the availability of high-density genetic maps that allow for detailed analysis of the genetic architecture of complex traits, anchoring physical contigs in genome sequencing projects and mapping genes of interest. Polyploidy and abundance of repetitive DNA complicate the analysis of genetic variation and the development of high-density genetic maps of the wheat genome. However, the advent of next generation sequencing provides efficient and cost effective tools for variant analysis even in this highly complex genome. Initially, next-generation sequencing was mostly used for the discovery of single-nucleotide polymorphisms (SNPs). During the past few years, several studies concentrated their efforts on the analysis of complexity-reduced fraction of the wheat genome (Trebbi *et al.* 2011) or cDNA (Lai *et al.* 2012; Trick *et al.* 2012). SNPs discovered in these datasets were then successfully used to develop genotyping assays based on GoldenGate (Akhunov *et al.* 2009; Chao *et al.* 2010), BeadExpress (Trebbi *et al.* 2011), KASPar (Allen *et al.* 2011), and Infinium platforms (Cavanagh *et al.* 2013).

With increases in the throughput of sequencing technologies, the approaches applied for genetic variation analysis start shifting from the usage of SNP-based genotyping assays to the direct sequencing of populations (Hamilton and Buell 2012). This latter approach allows for direct analysis of genetic variation reducing the effect of ascertainment bias caused by the SNP discovery process. The bias caused by the reference-guided variant discovery can now be solved by using multiple reference genomes (Gan *et al.* 2011). Depending on the genome size, re-sequencing is performed either on genomic DNA (Huang *et al.* 2009) or focused on sequencing of complexity-reduced DNA samples. Targeted approaches using the oligonucleotides designed to capture specific sequences of interest in the genome has been widely used in mammalian genome analysis (Bamshad *et al.* 2011) and were more recently developed on plants (Fu *et al.* 2010; Haun *et al.* 2011; Saintenac *et al.* 2011; Salmon *et al.* 2012; Winfield *et al.* 2012). Complexity reduction using restriction enzyme digests are based on digestion of genomic DNA by restriction enzymes and called RAD-seq, CroPs, or genotyping-by-sequencing (GBS) (Davey *et al.* 2011). Increasing capacities of sequencing technologies combined with the complexity reduction allow for multiplexing of 48 to 96 samples. The RAD-seq approach has been used for developing high-density maps of different species (Miller *et al.* 2007; Baird *et al.* 2008; Barchi *et al.* 2011; Davey *et al.* 2011; Bus *et al.* 2012). More recently, the GBS approach involving fewer steps than RAD-seq, was successfully applied for constructing genetic maps of crops with large genomes (Elshire *et al.* 2011; Poland *et al.* 2012). Although these early studies demonstrate the utility of complexity-reduced sequencing for genome analysis, detailed investigations are now required for understanding the limitations of low-coverage, next-generation sequence data for variant calling in the large wheat genome and developing approaches for mapping the genes of interest, ordering physical contigs, and performing association mapping.

One of the broadly used wheat reference mapping populations was developed by crossing genetically diverse synthetic wheat W7984 and cultivar Opata. A recombinant-inbred line population developed by crossing these parents was extensively used in gene mapping projects (Graingenes database: http://wheat.pw.usda.gov/GG2/index). This population was recently reconstructed (Sorrells *et al.* 2011) and genotyped using several approaches (Sorrells *et al.* 2011; Poland *et al.* 2012). Here, we developed two complexity-reduced libraries from the reconstructed SynOpDH population (Sorrells *et al.* 2011) and built a genetic map integrating more than 420,000 markers including gene-associated SNPs from the wheat 9K iSelect genotyping assay, GBS markers from three genomic libraries, diversity array technology (DArTs), and simple sequence repeat (SSR)/sequence-tagged site (STS). A bioinformatical pipeline for accurate variant calling in the GBS datasets generated for the polyploid wheat genome was developed. Using different combinations of restriction enzymes, we generated GBS datasets that showed small overlap thereby increasing the coverage of the wheat genome. Finally, we analyzed the distribution of these markers in the genic and intergenic regions of the wheat genome, and investigated their utility for gene mapping and ordering sequence contigs in genome sequencing projects.

## Materials and Methods

### Plant material

DNA was extracted from the 2-wk seedlings of the doubled-haploid Synthetic W7984 (Syn) × OpataM85 (Op) mapping population [henceforth, SynOpDH (Sorrells *et al.* 2011)] using the BioSprint 96 robot from QIAGEN. A total of 182 lines from the SynOpDH population were included into the experiment. DNA was quantified using the Quant-iT PicoGreen (Invitrogen) and concentrations were adjusted to 20 ng/ul.

### Complexity-reduced genomic libraries

Complexity-reduced genomic libraries were prepared using the two combinations of restriction endonucleases (RE): PstI (CTGCAG, methyl sensitive) with MseI (AATT, methylation-insensitive) and PstI with MluI (ACGCGT, methylation-sensitive). We used a set of 96 barcoded adapters (Supporting Information, File S1) with sticky ends complimentary to 3′ overhang created by PstI and MseI- or MluI-specific Y-adapters. Y-adapters were prepared by annealing a common primer 5′-CTCGGCATTCCTGCTGAACCGCTCTTCCGATCT-3′ with enzyme-specific primers 5′-taAGATCGGAAGAGCGGGGACTTTAAGC-3′ for MseI and 5′-cgcgAGATCGGAAGAGCGGGGACTTTAAGC-3′ for MluI. Annealing was performed in a thermal cycler in 50 mM NaCl, 10 mM Tris-HCl buffer (pH = 8.0) by slowly ramping down the temperature from 95° to 30° (1°/minute). After annealing, barcoded adapters were quantified using Quant-iT PicoGreen (Invitrogen) and diluted to a final concentration of 0.2 μM.

The library construction was performed in 96-well plates. Each plate included 90 doubled haploid lines and three replications of each parental line. The latter was used to increase the depth of sequence coverage to ensure reliable detection of polymorphisms between the parental lines. A total of 400 ng of genomic DNA was digested using 10 units of each RE in a 30-μL reaction volume for 3 hr at 37° followed by heat-inactivation at 80° for 20 min. The digested DNA samples were mixed with 0.6 pmoles of barcoded adapters and 8 μL of annealed MseI Y-adapter for the preparation of the PstI-MseI library or with 0.2 pmoles of barcoded adapters and 1 μL of annealed MluI Y-adapter for the preparation of the PstI-MluI library. Reaction mix was incubated with 6 units of T4 DNA ligase (NEB) at 22° for 2 hr and then enzyme was inactivated by heating at 65° for 20 min. Five microliters of DNA sample from each well in a plate was pooled, purified using QIAGEN column (QIAquick PCR Purification Kit), and eluted in a 50-μL volume. Libraries were amplified using 10 pmoles of polymerase chain reaction (PCR) primers 5′-AATGATACGGCGACCACCGAGATCTACACTCTTTCCCTACACGACGCTCTTCCGATCT-3′ and 5′-CAAGCAGAAGACGGCATACGAGATCGGTCTCGGCATTCCTGCTGAA-3′ in a 50-μL reaction mix using Taq 2X Master Mix from NEB. PCR cycling included 95° for 3 min. followed by 18 cycles of 95° for 30 sec, 62° for 10 sec, and 68° for 20 sec for the PstI-MseI library or 18 cycles of 95° for 30 sec, 62° for 20 sec, and 68° for 30 sec for the PstI-MluI library and terminated by a final extension at 72° for 5 min. Libraries were purified as previously described. The size distribution of DNA fragments in the genomic libraries and the presence of contaminating adaptor-dimer peaks were tested on Bioanalyzer. After estimating the average DNA fragment length (~240 bp), each library (pool of 92 individuals) was diluted to 10 nM concentration and sequenced on a single lane of Illumina HiSeq2000 flow-cell (100 bp single-end read run).

### SNP calling and mapping

A custom *perl* pipeline was developed to process and analyze complexity-reduced genomic sequence data (Figure S1). Reads were quality trimmed from each side by removing bases with phred33 quality score <15. Reads with >20% of bases having quality score <15 were also discarded. Each read was tested for the presence of a PstI site following the barcode sequences, which were used to assign reads to individual lines. After quality filtering, in average, 77% of all reads could be assigned to individual lines.

Opata M85 and Synthetic W7984 reads were grouped using CD-HIT (Fu *et al.* 2012) into a nonoverlapping set of unique clusters. Clustering was performed using 96% similarity threshold that permits the alignment of divergent homeologoues sequences from different wheat subgenomes. The nonredundant Op and Syn clusters that differ from each other by no more than three mismatches were used as a reference sequence. Illumina reads were aligned uniquely to the reference using *bowtie* (Langmead *et al.* 2009) with settings tolerating three mismatches in aligned reads (parameter settings: −v 3 –k 1). Variant discovery and genotype calling were performed using SAMTools (Li *et al.* 2009).

To reduce the number of false-positive results, postprocessing filters were applied to genotype calling data. First, variable sites showing more than two states or observed only in the mapping progeny but not between the parental lines have been removed. To reduce the number of falsely discarded variable sites, for <2% of individuals in the mapping progeny we permitted to have basecalls different from that of the parental lines. These genotypes were recorded as missing data. Finally, variable positions with <80% missing data and fitting 2/3 allele coverage ratio were recorded as a genotyping matrix that was used to create a genetic map.

The two genetic map construction approaches were implemented in a pipeline. The first approach used a reference map constructed using either an alternative marker system or a high quality subset of GBS markers (henceforth, bin-mapping approach). The second approach directly used high-quality GBS markers carrying SNPs to build a genetic map (henceforth, *de novo* mapping approach). Because reads from different wheat genomes are aligned together to the same reference sequence, the probability of recovering an allele from the genome carrying the SNP is twice less than the probability of observing the corresponding nucleotide on the other homeologues. Therefore, regions with low coverage may result in SNP miscalling and inflate the genotyping error rate. Cosegregation of allelic variants with the markers on the reference map can effectively be used for the removal of erroneously called variants and mapping markers with the high fraction of missing data. SNPs showing ≤10% recombination fraction with at least one marker on the reference map were integrated into the map. To calculate recombination frequencies, the number of parental (AA and BB) and recombinant gametes (AB and BA) between a GBS marker and a reference marker were counted. GBS markers linked with reference markers mapped to different chromosomes were excluded from the final map. If a GBS marker was associated with more than one cosegregating marker on the reference map, it was mapped using the marker with which it shared the smallest number of recombination events. If a GBS marker cosegregated with more than one marker with which it shared the same number of recombination events, it was placed in the top part of the recombination interval delimited by these markers. On the final map each GBS marker was mapped to a recombination interval defined by two markers of the reference genetic map. The genotyping matrix for *de novo* mapping was generated by selecting high-quality GBS markers carrying SNPs that had only one variant in each parental line, and showed ≤20% of missing and ≤5% of heterozygous genotypes.

To call presence/absence (PA) variants, Op and Syn reads were clustered using 100% similarity threshold. Nonredundant reference reads were used to align reads from each DNA sample permitting only one mismatch. Mapping was performed using only the bin-mapping approach for markers having “presence” allele calls in at least 10 individuals in the SynOp progeny. The significance of association between the “presence” allele of a PA variant and reference marker alleles was tested using the binomial distribution. Markers passing the significance threshold of *P* < 0.001 and showing >10% recombination fraction with the reference markers were removed.

### Genetic maps construction

Genotyping data previously generated for the SynOpDH population using DArT, SSRs (Sorrells *et al.* 2011), and 9K iSelect (Cavanagh *et al.* 2013) were integrated to create a high-density reference map. Genetic maps were built using Carthagene (de Givry *et al.* 2005). Linkage groups were constructed using the LOD score of 6 and the maximum distance of 20 cM. To create a *de novo* map from GBS-associated SNPs, linkage group were build using the LOD score of 8 and the maximum distance of 20 cM. Ordering was performed using the default parameters. The SynOpDH integrated genetic map is available for download from the GrainGenes website (http://wheat.pw.usda.gov/ggpages/map_summary.html).

## Results

### Variant calling and genotyping

To saturate the wheat genome with genetic markers, we constructed two genomic libraries generated using the methylation sensitive RE PstI combined with either the six base-pair cutting RE MluI (ACGCGT, methylation sensitive) or the four base-pair cutting RE MseI (AATT, methylation insensitive). A total of 92 individuals were digested with the restriction enzyme pair PstI–MluI, and 182 individuals were used to prepare the PstI-MseI complexity reduced library. To increase the number of markers, raw PstI-MspI sequences generated from the same population were downloaded from the NCBI database (Poland *et al.* 2012) and processed using the bioinformatical pipeline described here. With a total of 408 million reads for these three libraries, we obtained an average of 0.81 million reads per individual for PstI-MseI library, 0.8 million reads per individual for PstI–MluI library, and 0.93 million reads per individual for PstI-MspI library. To increase the accuracy of variant calling, nearly three times more sequence data were generated for each parental DNA sample (Table 1).

**Table 1:** Summary of next-generation sequence data used for variant analysis

Library	No. Illumina Reads, ×10^6^	Average No. Reads per Individual, ×10^6^	No. Reads for Each Parent (Syn/Op), ×10^6^	No. Clusters at 100% Similarity (Syn/Op), ×10^6^	No. Clusters at 96% Similarity (Syn/Op), ×10^6^
PstI-MseI	155	0.81	4.3/4.5	1.19/1.16	0.69/0.65
PstI-MluI	78	0.80	2.7/2.8	0.24/0.20	0.11/0.07
PstI-MspI*^a^*	175	0.93	3.6/4.3	1.37/1.57	0.72/0.78

a Data downloaded from the NCBI database (Poland *et al.* 2012).

Low-coverage sequencing of complexity-reduced libraries resulted in a large proportion of missing data in each sample. This factor, combined with polyploidy and high repetitive DNA content significantly complicates the analyses of next-generation datasets generated for the wheat genome. To overcome these complexities, we used a pipeline developed for variant calling (SNP and presence/absence) and mapping in the polyploid wheat genome (Figure S1).

To maximize the usage of available sequence data and effectively separate sequence reads originating from duplicated genomic regions, we performed read trimming based on the base quality rather than using the fixed read length (64 bp) previously applied for analyzing GBS data in wheat and barley (Poland *et al.* 2012). To identify polymorphisms, the Syn and Op reads were clustered and used as a reference to align reads from each individual of the SynOpDH population. We have optimized similarity thresholds for read clustering separately for SNP and PA variant calling followed by removal of redundant genotype calls.

The PA variation discovery was performed using clusters generated using the 100% similarity threshold and allowing for one mismatch during the alignment step (Table 1). This approach can effectively identify PA variation even in the presence of divergent nonallelic duplicated DNA fragments in the genome. However, it can inflate the false PA variant detection rate if the parental alleles are highly diverged. These divergent alleles instead of being mapped as a single codominant marker will be mapped to the same position as two different dominat PA loci. It will remain, however, difficult to differentiate the divergent parental alleles from duplicated loci present in the same genomic location.

The reference sequence for SNP discovery was generated by clustering reads using the 96% similarity threshold. At the alignment step, three mismatches were permitted. These clustering and read mapping parameters increase the number of identified SNPs by allowing for the alignment of divergent allelic variants and duplicated homeologous sequences.

### Development of high-density wheat reference genetic map

The reference genetic map was constructed by integrating several datasets. We used SNP data generated for 178 individuals from the SynOpDH population using the wheat 9K iSelect assay (Cavanagh *et al.* 2013). A high-quality reference map containing 4209 loci was built by combining the 2740 gene-associated SNPs from this assay and the previously mapped 118 SSR/STS and 1351 DArTs markers (Sorrells *et al.* 2011). Using the bin-mapping approach implemented in the pipeline, we mapped 33,885, 1639, and 25,153 SNP-containing GBS tags from the PstI-MseI, PstI-MluI, and PstI-MspI libraries, respectively (Table 2). The average bin size associated with each mapped SNP was 1 cM, and the genetic locations of 62% of SNPs could not be resolved in the SynOpDH population. In average, 37% of genotype calls per marker were absent and 62% of markers had less than 50% of missing data. A total of 25,261, 1247, and 20,064 unique loci were mapped in the PstI-MseI, PstI-MluI, and PstI-MspI libraries, respectively. It is noteworthy that 98% of SNPs originating from the same reference cluster were mapped to the same genetic position. Only 205 (0.6%) and 3036 (9%) SNPs discovered in the PstI-MseI library were shared with the SNPs discovered in the PstI-MluI and PstI-MspI libraries, respectively.

**Table 2:** Summary of genetically mapped PstI tags

Library	No. Tags with SNPs	No. Tags with PAV	No. Tags Used for *de novo* map (with SNPs)
PstI-MseI	33,885	167,049	2703
PstI-MluI	1639	14,255	271
PstI-MspI	25,153	216,027	907

PAV, presence absence variation; SNP, single-nucleotide polymorphism.

We mapped 167,049, 14,255, and 216,027 PA loci from the PstI-MseI, PstI-MluI, and PstI-MspI libraries, respectively (Table 2). The average size of the recombination bins on the reference map associated with the PA markers was 10.2 cM. The increased bin size compared to that obtained for SNPs is likely due to the smaller number of individuals used for PA variant mapping (see *Material and Methods*). The limited number of recombination events in these lines can increase the number of reference loci associated with a mapped PA marker. We identified up to 1482 (0.9%) PA markers shared between PstI-MseI and PstI-MluI genomic libraries and up to 22,073 (13.2%) markers shared between PstI-MseI and PstI-MspI libraries. The small proportion of shared SNP and PA variants between libraries suggests that the usage of different RE is an efficient approach to increase the coverage of the wheat genome.

To test the efficiency and accuracy of the applied mapping strategy, we first compared the map locations of SNPs shared between the 9K iSelect SNP genotyping assay (Cavanagh *et al.* 2013) and the genomic libraries. The map locations of thirty-seven shared SNPs were nearly identical on both genetic maps and differed by only an average of 0.57 cM (σ = 1.02). Furthermore, we compared the genetic positions of SNPs and PA variants shared among different genomic libraries. Of 3467 shared SNPs, 3463 (~100%) mapped to the same chromosome with a mean genetic position difference of 1.16 cM (σ = 2,04). Of 19,945 PA variants shared between the PstI-MseI and PstI-MspI libraries, 19,318 (97%) were mapped to the same chromosome with a mean difference of 3.25 cM (σ = 4.7). Taken together, these results suggest that the variant calling and genetic mapping strategy implemented in the pipeline can generate accurate maps of the wheat genome.

To create the final high-density reference map, we cross-compared all GBS sequences to remove redundancy. Because PA variant analysis can sometimes result in the identification of SNPs as dominant markers, we removed redundant PA markers if they were previously identified as SNP-containing markers. The total number of GBS-derived markers mapped to the wheat A, B, and D genomes was 137,604, 179,802, and 99,450, respectively. As expected, more markers were mapped to the largest chromosome (Chromosome 3B: 32,060), whereas the smallest number of markers (6865) was mapped to chromosome 4D, the smallest wheat chromosome (Table S1). The number of PstI tags generated using two methylation sensitive hexanucleotide cutters (PstI and MluI) was substantially lower than that obtained for two other RE combinations. No significant library-specific bias was observed for the total number of markers mapped per individual wheat chromosome (Table S1). To estimate the redundancy of PA variants between different libraries, we calculated the percentage of highly similar (>96%) PA variants in 10 cM intervals along the wheat chromosomes. We identified that in average less than 11% of PA markers are duplicated. In total, we mapped 421,065 markers on the SynOpDH population including 1351 DArTs, 118 SSR/STS, 59,967 SNP, and 359,629 PA markers (Table S1) achieving an average marker density of 1 marker per 380 kb for the 16-Gb wheat genome.

### Accuracy of a *de novo* genetic map

The accuracy of a *de novo* GBS marker map was assessed by comparing it with the high-density reference map developed using the 9K iSelect genotyping assay (Cavanagh *et al.* 2013). We used 2703 high-quality SNPs selected from the PstI-MseI library to create a *de novo* genetic map. Using stringent grouping parameters (distance >20 cM and LOD value >8), we obtained 23 linkage groups. More markers were mapped in the B genome (1185) than in the A (781) and D genomes (735) (Table S2). In average, 129 markers were mapped per chromosome. The total length of the *de novo* map was increased by a factor of 1.6 compared with that of the high-density reference map. This increase is most likely associated with genotyping errors in the GBS dataset (Figure S2). Surprisingly, the distal parts of short arm 1D and 5A as well as the distal regions of long arm 2B, 4A, 4D, and 7B were missing in the *de novo* map. However, 23 markers present in the *de novo* map were not included in the map constructed using the bin-mapping approach. These markers are present in the regions not adequately covered by the reference markers. Nevertheless, the order of markers between the maps developed using the bin-mapping and the *de novo* approach was mostly consistent (Figure S2). When the *de novo* map was used as a reference for bin-mapping strategy, we were able to position 35,070 SNPs and 194,752 PA markers identified by the pipeline in the PstI-MseI library. Of these markers, 32,945 SNPs (93.9%) were shared with the PstI-MseI library SNPs mapped using the reference map build from the wheat 9K iSelect assay.

### Distribution of PstI-associated sequences in the wheat genome

**Figure 1:**
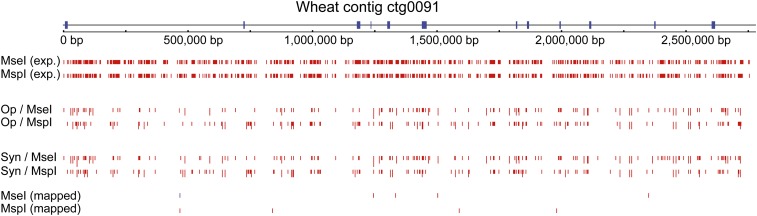
Distribution of PstI-associated sequence reads along the 3 Mb-long wheat contig. The positions of expected (exp.), observed and genetically mapped PstI tags along wheat contig ctg0091 (Choulet *et al.* 2010) are shown for the parents (Syn and Op) of the SynOpDH mapping population. There are 7 PA and 1 SNP variants are mapped (red and blue vertical lines for PA and SNP variants, respectively, on the MseI (mapped) and MspI (mapped) tracks). The line length reflects the frequency of tags in a library. The locations of annotated genes on the wheat contig are shown as blue bars.

The distribution of PstI-associated sequence reads in the wheat genome was investigated using the 18.2 Mb of annotated genomic regions scattered along chromosome 3B (Choulet *et al.* 2010). The expected density of PstI-MseI (3.3 tags per kb) and PstI-MspI (3.4 tags per kb) tags in these contigs was similar, whereas the expected density of PstI-MluI tags (62.8 tags per kb) was much lower. Using perfect match alignment, we showed that approximatly 30% of expected PstI-MseI and PstI-MspI tags were present in the genomic libraries of at least one parental line and 45% of tags were present in both parental lines (Figure 1). Only 15% of the expected PstI-MluI tags were found in the PstI-MluI genomic library. However, 20% of GBS tags that match the chromosome 3B contigs were not expected by *in silico* digestion due to modifications at the PstI sites. The frequency of modified cut sites with 1-, 2-, 3-, 4-, 5-, and 6-bp differences was 0.27, 0.16, 0.07, 0.15, 0.2, and 0.15, respectively.

Next, we estimated the proportion of expected PstI tags that were genetically mapped using the SynOpDH population. The comparison of the expected PstI tags with the mapped genomic sequences was performed allowing either no mismatch or three mismatches (similar to alignment stringency used for SNP discovery). Out of 10,970 expected PstI tags in the chromosome 3B contigs, 2.8% were genetically mapped. Of 21 genetically mapped PstI tags that harbor SNPs with one of the variants having a perfect match to the chromosome 3B contigs, 20 (95%) were genetically mapped to chromosome 3B. Using lower alignment stringency (3 mismatches), we identified 302 PstI tags that match the chromosome 3B contigs. Among them, only 101 (33%) were genetically mapped to chromosome 3B. It is noteworthy that 92% of these genetically mapped tags were expected by *in silico* digestion. Out of 272 PstI tags showing PA variation and matching perfectly the chromosome 3B contigs, 218 (80%) were mapped to chromosome 3B with 208 (76%) mapping to the correct genetic position.

Interestingly, the proportion of correctly mapped PstI tags having perfect match to chromosome 3B was highest for contig 0011b, which also shows the lowest content of transposable elements (Table S3). To understand the impact of transposable elements on GBS tag mapping, we estimated the mean frequency of PA markers that perfectly match the chromosome 3B contigs and map genetically either to chromosome 3B or other chromosomes. The latter class of PstI tags most likely originates from highly repetitive sequences distributed across the wheat genome. Mean genome-wide frequency of PstI tags mapped genetically to chromosome 3B (three tags) was substantially lower than the mean frequency of tags (287 tags) mapped to other chromosomes. This result suggests that the majority of GBS tags showing PA variation originate from highly repetitive fraction of the wheat genome. Consistently, in the PstI-MseI library of the Op parental line, more than 10% of reads are found in highly covered tags (depth > 100 reads) with the maximum depth of coverage of 70,400 reads. These data suggest that the application of PA variation for genome analysis and mapping will have some limitations. The alignment of PA variants to a reference genome, depending on its complexity, will detect a significant number of highly similar hits distributed across different chromosomes thereby limiting the utility of PA markers for the precise mapping of associated traits or ordering sequence contigs generated in genome sequencing projects. The limited ability to reliably call the “absence” alleles in low-coverage GBS datasets can inflate the number of false-positive variants and requires that the PA markers are mapped using the bin-mapping approach thereby restricting the resolution of a generated map to genetic distances between the markers on a reference map. Moreover, because PA variant detection depends on the alignment stringency, the highly divergent GBS tags representing different alleles of the same locus can be mistaken for two independent PA loci.

Finally, the chromosome 3B contig sequences were used to investigate the effect of chromosome location on the distribution of PstI tag-associated polymorphisms. The level of diversity was higher in the contigs located in the distal chromosomal bins (Table S3). Consistent with the results of whole genome comparison, the populations of PstI tags having hits to the wheat contigs showed small overlap among different libraries. On contigs 0011b and 0954b, out of 250 genetically mapped markers, only 20 (8%) were shared between the PstI-MseI and PstI-MseI libraries.

### Resource for high-resolution gene mapping

**Figure 2:**
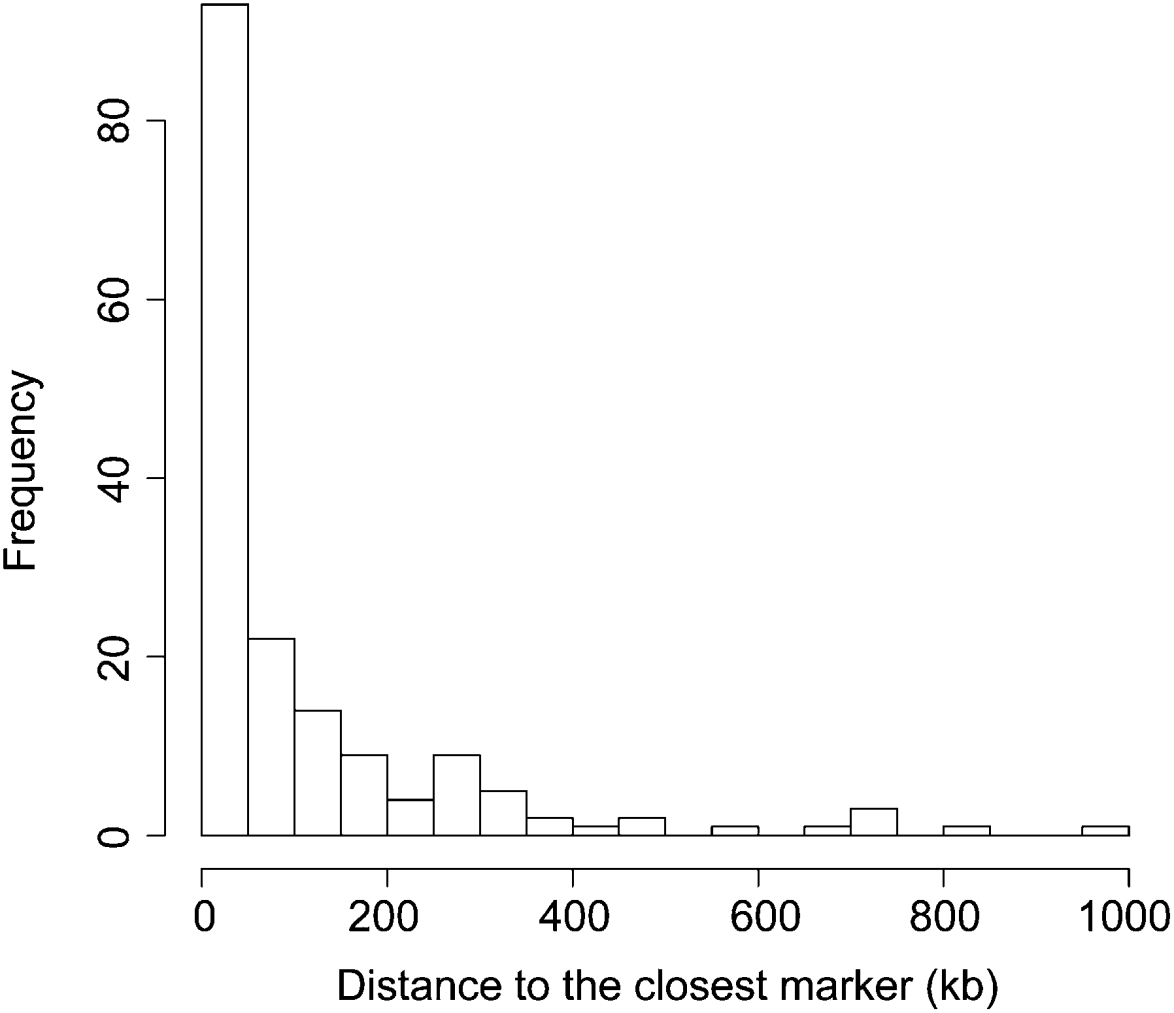
Distance distribution between genes and the closest markers mapped in the SynOpDH population. A total of 168 gene models extracted from the annotated wheat contigs (Choulet *et al.* 2010) were used to assess the distance from the 5′end of each CDS to the closest genetically mapped PstI tag.

Because of the sensitivity of the PstI to DNA methylation, its cutting sites are preferentially located in low methylated genomic regions usually enriched for gene coding sequences (Fellers 2008). Using 168 genes identified on the 3B contigs, we estimated that the average physical distance between a gene and the closest genetically mapped GBS marker is 108 kb. Moreover, 55% of genes had a marker located at <50 kb, and 14% of genes contained a mapped PstI tag within the genic region (Figure 2).

The distribution of PstI tags relative to coding regions was further investigated using 19 sequence contigs scattered throughout the wheat genome and harboring genes underlying important agronomic traits (Table 3). For each contig, we estimated the number of PstI tags genetically mapped to the expected chromosomal location. We were able to identify GBS markers for fourteen out of the nineteen loci studied. Three of these genes, *Lr*21, *Lr*34, and *Sr*2, conferring resistance to fungal pathogens, were previously mapped using SSR/STS markers on the SynOpDH population (Sorrells *et al.* 2011). We identified six and twenty PstI tags that physically map to the *Lr*34 and *Sr*2 loci, respectively, and cosegregate with previously identified diagnostic markers. The closest SNP cosegregating with the *Lr*34 gene was located within 24 Kb. Two of five PstI tags physically mapped to the *Lr*21 locus were located within the resistance gene. The proximal location of *Vrn*1 (Yan *et al.* 2003) and *Vrn*3 (Yan *et al.* 2006) loci might explain the lack of linked markers due to reduced mapped marker density in the proximal chromosomal regions. Among the analyzed loci, the average marker density was one marker every 30 kb and the average distance between a cloned gene and the closest marker was 41.5 kb (Table 3).

**Table 3:** Distance from wheat genes underlying important agronomic traits to closest marker

Gene	Chr.	Contig Size, kb	Total No. Markers	Closest Marker to the Cloned Gene, kb	GenBank ID	References
*Lr21*	1DS	28	4	0	AF532104	(Huang *et al.* 2003)
*Lr34*	7DS	207	5	24	FJ436983	(Wicker e*t l*. 2009)
*Sr2*	3BS	3109	142	NA*^a^*	FN564434	(Paux *et al.* 2008)
*Lr1*	5DL	138	26	0	EF567062	(Cloutier *et al.* 2007)
*Lr10*	1AS	187	1	113	AY663391	(Isidore *et al.* 2005)
*Yr36*	6BS	314	1	39	EU835198	(Fu *et al.* 2009)
*Vrn1*	5AL	133	0	−	AY188331	(Yan *et al.* 2003)
*Vrn2*	5AL	439	1	102	AY485644	(Yan *et al.* 2004)
*Vrn3*	7BS	104	0	−	DQ900686	(Yan *et al.* 2006)
*Q*	5AL	109,149	1	0.9	AY914082, JF701614	(Faris *et al.* 2003;Simons *et al.* 2006)
*Gpc-B1*	6BS	245	16	2.3	DQ871219	(Uauy *et al.* 2006)
*Rht*	4DS	207	1	148	HQ435325	(Duan *et al.* 2012)
*Glu-1*	1AL	292	1	89	DQ537335	(Gu *et al.* 2006)
*Glu-1*	1BL	206	0	−	DQ537336	(Gu *et al.* 2006)
*Glu-1*	1DL	152	3	6	DQ537337	(Gu *et al.* 2006)
*Glu-3*	1AS	70	7	3	FJ447464	Direct submission
*Glu-3*	1BS	72	0	−	FJ447463	Direct submission
*Glu-3*	1DS	100	8	54	FJ447462	Direct submission
*Ha*	5DS	94	8	1	CR626934	(Chantret *et al.* 2005)

a *Sr2* gene is absent in *T. aestivum* cv. Chinese Spring. NA, not available.

A substantial number of PstI tags showed similarity to known wheat genes. For example, ten markers corresponding to the *Pm3b* gene (Yahiaoui *et al.* 2004) and two markers representing gene *Ppd-D1* (Beales *et al.* 2007) were present on our map. Genes encoding the α-gliadin storage, *Lrk10*, viviparous, sucrose synthase, granule-bound starch synthase proteins were genetically mapped (Table 4) to positions similar to those reported previously (see GrainGenes database at wheat.pw.usda.gov). Seven PstI tags orthologous to barley RGA sequences (Madsen *et al.* 2003) were mapped to chromosomes 2D, 3B, 4A, 5A, 5B, 5D, and 7B. Moreover, GBS markers matching the wheat RGA sequences were mapped to chromosomes 1D, 2A, 3A, 3D, 4B, 5D, and 7D.

**Table 4:** List of PstI tags showing similarity to known genes

Marker Name	Chr.	Genetic Interval, cM	Matches in NCBI Database
M6_692227_PstIMspI_PA	2D	22.5-26.6	DQ885766, *T. aestivum* pseudo-response regulator (PRR) gene
Opata_3519656_48_PstIMspI_SNP	1A	0	AY325736, *T. aestivum* powdery mildew resistance protein PM3b (Pm3) gene
Opata_2857704_63_PstIMspI_SNP	6A	23.1	U08287, *T. aestivum* alpha-gliadin gene
Opata_54442_67_PstIMseI_SNP	1A	18.6	U76215, *T. aestivum* NBS-LRR type protein
Opata_464857_12_PstIMseI_SNP	3A	58.1	AJ400712, *T. aestivum* vp1A gene for VIVIPAROUS1
Opata_292667_17_PstIMseI_SNP	7B	35.4	M26671 *T. aestivum* sucrose synthase type 1
Opata_67370_7_PstIMseI_SNP	7A	8.7	AF113843, *T. aestivum* granule-bound starch synthase precursor (Wx-A1)
Opata_129253_76_PstIMseI_SNP	5D	62.3	AB630961, *Ae. tauschii* Vrn-D1 gene for MADS-box protein
Opata_2424013_69_PstIMspI_SNP	5B	66.6	JN817431, *T. durum* Vrn-B1 (VRN-B1) gene
M6_320736_PstIMseI_PA	2D	48.7−72.6	AF032683, *H. vulgare* NBS-LRR type resistance protein (b5) gene
M6_335919_PstIMspI_PA	5D	107	AJ507089, *H. vulgare* NBS-LRR protein
M6_57814_PstIMseI_PA	7B	0	AJ507089, *H. vulgare* NBS-LRR protein
Opata_2229181_41_PstIMspI_SNP	5B	104.9	AJ507089, *H. vulgare* NBS-LRR protein
Opata_96382_PstIMspI_PA	5A	103.4-113.7	AJ507092, *H. vulgare* NBS-LRR protein
Opata_786282_PstIMseI_PA	3B	66.7-81.1	AJ507098, *H. vulgare* NBS-LRR protein
Opata_780498_60_PstIMspI_SNP	5D	70	AF320845, *T. aestivum* NBS-LRR protein
Opata_1722669_54_PstIMspI_SNP	7D	59	AJ420959, *T. turgidum* cre3 gene
Opata_740733_PstIMseI_PA	4B	40.9	AJ488510, *T. turgidum* RGA4 protein
Opata_2018933_82_PstIMseI_SNP	1D	22.5	AF538040, *T. aestivum* Mla-like protein
Opata_3999958_PstIMseI_PA	2A	74.2-78.2	GU356591, *T. aestivum* NBS-LRR protein
M6_1454012_PstIMseI_PA	2A	87.3-96.5	GU067479, *Thinopyrum intermedium* NBS-LRR resistance protein
Opata_1286128_20_PstIMspI_SNP	3D	72.2	JF957106, *T. aestivum* isolate putative non-TIR-NBS-LRR resistance gene analog gene
Opata_1956156_11_PstIMseI_SNP	7D	115.5-115.8	DQ205351, *T. aestivum* disease resistance protein
Opata_4189631_PstIMspI_PA	3A	102.5	DQ241562, *T. aestivum* powdery mildew resistance protein
Opata_862901_PstIMseI_PA	4A	92.4-104.9	JN818648, *H. ordeum* vulgare NBS-LRR resistance-like protein (RGH-2)

Finally, we mapped loci controlling the seed color segregating in the SynOpDH population (Sorrells *et al.* 2011). This phenotype is controlled by genes encoding the MYB transcription factors mapped to the long arm of chromosomes 3A, 3B, and 3D (Himi *et al.* 2011). In the SynOpDH, both parents have red seeds and show 3 red/1 white segregation ratio for seed color suggesting that this trait in the parental lines can be controlled by two loci with a dominant red seed color allele. Then, the individuals with white seeds should carry white seed color allele on two loci, one originating from Op and the other one from Syn parents. Among individuals with white seeds, we found that the locus on chromosome 3B is fixed for the Syn allele and the locus on chromosome 3D is fixed for the Op allele. A total of 1437 and 1022 markers on the SynOpDH map cosegregated with the 3B and the 3D loci, respectively. The sequence of one of the co-segregating PstI tags matched the *Tamyb*10-D1 gene identified by Himi *et al.* (2011). Our results suggest that on the SynOpDH map we can expect to find several markers located within several tens of kilobases from a gene of interest.

### Anchoring shotgun sequence assemblies in genome sequencing projects

A large number of mapped PstI tags can effectively be used for contig anchoring in genome sequencing projects. To test the utility of the developed reference map for ordering sequence contigs we compared PstI tags with the shotgun sequence assemblies generated for flow-sorted wheat chromosome 3A (Akhunov *et al.* 2013). Of 18,895 PstI tags genetically mapped to chromosome 3A, 3664 could be aligned to 2561 contigs and 1541 scaffolds (4% of the total number of contigs/scaffolds) (File S2). These markers are represented by 985, 634, and 44 SNP-harboring PstI tags and 1010, 951, and 40 PA variants from the PstI-MseI, PstI-MspI, and PstI-MluI libraries, respectively. Compared with the entire chromosome 3A shotgun assembly, mapped contigs were significantly enriched for genic sequences. Of 3664 mapped PstI tags, 29% were mapped to contigs/scaffolds harboring annotated genes consistent with increased density of PstI sites in close proximity to genic regions. Because of the low resolution of the reference genetic map, the anchored scaffolds could be allocated to 65 recombination intervals on chromosome 3A.

## Discussion

In this study, we developed an integrated reference genetic map of 421,065 markers including about 60,000 SNPs, and demonstrated its utility for mapping genes of agronomic importance in wheat, and for anchoring physical and genetic maps in genome sequencing projects. To link the reference map with previously developed resources, 118 SSR/STS and 1351 DArTs markers broadly used in wheat genetic studies have been included.

Our bioinformatical pipeline was shown to be effective for variant calling in a GBS dataset generated for a polyploid genome. To compare the effect of polymorphism detection strategy on variant calling results, we compared SNP sets discovered in the PstI-MspI library using different approaches. Between the previously identified set of 19,720 SNPs (Poland *et al.* 2012) and 25,153 SNPs identified in our study, we found only 8891 shared SNPs. Among nonshared SNPs, 6719 where identified in the 3′ region of the reads located after base 64. In addition, a total of 4940 PstI tags that were previously shown to harbor SNPs (Poland *et al.* 2012) were mapped as PA variants in our dataset. These results can potentially be explained by two factors. First, read trimming using base quality criteria applied in our pipeline increased the length of analyzed sequence adjacent to PstI sites (in average 90 bp), thereby increasing the mapping accuracy in the highly duplicated genome and allowing us to perform SNP detection in larger genomic regions. Second, the stringency of read mapping in the pipeline was adjusted to permit homeologous copies of genes to align to the same reference also increasing the number of called SNP variants. Overall, these two variant calling approaches might be complementary, since they capture different features in the complex next-generation sequencing dataset. Further optimization of alignment and variant calling algorithms along with the usage of the wheat genome sequence as reference for read mapping can increase the number and accuracy of called variants.

We demonstrated that by using several restriction enzymes paired with PstI we increase the density of markers across the wheat genome. The minor overlap observed between the SNP and PA sets discovered using different RE pairs apparently results from both the differences in the distribution of restriction sites and the low coverage achieved for complexity reduced libraries. Since the expected overlap between the sets of tags generated by the PstI-MspI and PstI-MseI *in silico* digestion of wheat DNA sequences was 70%, the combination of RE pairs can only partially (up to 30%) explain differences between the sets of PstI tags in the libraries. Comparison of the two sets of PstI tags that were generated for the same parent (Op) in two independent DNA sample pools (PstI-MseI library) and including 1.4 and 3.1 million reads showed that only 50% of PstI tags in the first library overlap with those in the second library. Due to the low coverage, the polymorphism analysis of these two technical replicates would produce only partially overlapping SNP and PA sets. The depth of sequence coverage per individual is, therefore, a major factor to consider for achieving better reproducibility. In the 18.2 Mb sequence of chromosome 3B (Choulet *et al.* 2010), we identified 5455 *in silico* PstI-MseI fragments with sizes ranging from 30 to 1000 bp, which are similar to sizes observed in the genomic library profile. By extrapolating this estimate to the wheat genome we obtain 5.1 million expected PstI-MseI fragments. Therefore, the actual number of unique PstI tags (~1.2 million) obtained for Syn and Op parents in the genomic library represent only 20% of expected tags without considering methylation. However, when we used 155 million reads from all the individuals of the PstI-MseI library to estimate the number of unique PstI tags as a function of the number of reads included into the analysis, we observed nearly linear relationship between these two parameters (R^2^ = 0.96). We did not reach a plateau in the number of unique PstI tags in the dataset, expected when all PstI tags in genome are sequenced. Several factors can explain this observation: (1) PstI tags present in the sample of the chromosome 3B contigs are not representative of whole genome; (2) small overlap between the PstI sites identified in the Syn and Op parents can increase the total number of unique tags in the analyzed dataset; (3) the presence of highly repetitive PstI-harboring sequences; and (4) the presence of sequencing errors.

The depth of coverage per sample can be optimized using several approaches, one of which is the selection of RE pairs. As expected, we observed the lower level of missing data per individual (an average 14%) for tags generated from the PstI-MluI library compared with that from the PstI-MseI library (an average 44%). An alternative approach to reduce sample complexity further is to use selective primers for PCR amplification as was shown recently in the 2b-RAD-seq protocol (Wang *et al.* 2012). The same strategy applied to cDNA (Davey *et al.* 2011) will also remove most of the highly abundant tags at the same time limiting polymorphism discovery to genes.

The high-density SynOpDH map was shown to be an effective tool for mapping genes with many markers located either in close proximity to or within the genes of interest. Moreover, integration of SSR/STS and DArT markers into the reference map allows for linking thousands of SNPs and PstI tags with previously mapped genes and QTL providing resources for accelerated positional gene cloning in wheat. Once marker-trait associations are established, PstI tags can be converted to genotyping assays for application in breeding or high-resolution mapping. The efficiency of SNP conversion for complexity reduced libraries varied significantly depending on the complexity of studied genomes and approaches used for genomic library preparation and genotyping (Hyten *et al.* 2010; Mammadov *et al.* 2010; Barchi *et al.* 2011; Trebbi *et al.* 2011; Scaglione *et al.* 2012). For eggplant (Barchi *et al.* 2011) and soybean (Hyten *et al.* 2010), conversion rates were 89% and 81.6%, respectively. The conversion rate was lower for more complex genomes of maize (63%, (Mammadov *et al.* 2010) and wheat (35.8%, (Trebbi *et al.* 2011). Lower conversion rates in these two studies can be largely explained by the location of SNPs within repetitive sequences. The development of assays for highly repetitive genomes, such as wheat, requires additional filtering to remove abundant sequences by comparing against either databases of repetitive elements or by counting the frequency of sequence motifs (k-mers) in the genome (Kurtz *et al.* 2008).

The high-density SynOpDH map can also serve as a powerful tool for ordering contigs in whole genome sequencing projects. Hundreds of thousands of sequence tags can be easily compared with the contig assemblies for ordering along the chromosomes. However, these analyses should take into account the repetitive fraction of PstI tags originating from highly abundant genomic sequences. As we have shown here, 20% of GBS tags that have a perfect hit to 3B contigs were not expected by *in silico* digestion. Moreover, from 5 to 25 percent of GBS tags present on 3B contigs did not map to the expected genomic position on chromosome 3B. This result can be explained by the high frequency of GBS tags originating in the duplicated regions of the wheat genome. Even though these GBS sequences are present on chromosome 3B they map to a different location in the genome that harbor a duplicated GBS tag polymorphic between the parents of the SynOp mapping population.

In addition, it is currently not clear to what extent the bias in the distribution of PstI sites across genome caused by preferential location in proximity to genes can affect the final genome assembly. In the chromosome 3A shotgun sequence assemblies, the fraction of gene-harboring contigs among those that have hits with the PstI tags was greater (29%) than in the entire dataset. However, since sequencing of complex genomes includes the development of BAC-based minimum tiling path followed by its sequencing, we should expect a large number of hits to PstI tags within each contig, as was shown here by analyzing the chromosome 3B contigs.

In conclusion, the SynOpDH high-density map provides a large number of markers for wheat genome analysis and gene mapping. Since the reference map includes SSR/STS and DArT markers broadly used in the gene/QTL mapping projects, it is now quite straightforward to identify a large set of PstI tags linked with the region of interest and perform high-resolution gene mapping. It is important to emphasize that the analysis of this highly complex data was greatly facilitated by the usage of a bi-parental population that allowed us to separate true variants from errors based on the expected segregation in the progeny. Among 87,239 potential SNPs identified in the SynOpDH population, only 33,885 (39%) showed the expected allelic ratio and segregated with reference markers. This result suggests that 60% of identified variants are not linked with markers on the reference map used in the bin-mapping approach. Partially, unmapped SNPs can be explained by the presence of gaps in the reference map. However, because the number of recombination events in the F1-derived SynOp DH population is small and recombination intervals can be tagged using a relatively small number of markers, the most likely explanation for such a high proportion of unmapped SNPs is the high variant miscall rate. SNP discovery in wheat is complicated by allopolyploidy resulting from the two consecutive whole-genome duplication events and high repetitive DNA content. In the GBS dataset, due to the low coverage, we might falsely call SNPs at the sites of divergence between the homeologous genomes or duplicated genomic segments. These SNPs can still show the expected 1:1 allelic ratio in the mapping population, but will be randomly associated with markers on the reference genetic map. Considering such a high false-positive rate, application of this strategy for gene mapping in the populations of unrelated wheat accessions (association mapping) will be challenging and would require either increasing the depth of sequence coverage to increase genotype calling accuracy or the development of efficient imputation approaches based on the high-resolution haplotype map of the wheat genome.

## Supplementary Material

Supporting Information
