# Supplementary material for: Sequence-Based Mapping of the Polyploid Wheat Genome
Source: G3 (Bethesda). 2013 Jul 1;3(7):1105–14. doi: 10.1534/g3.113.005819 (PMC3704239; doi:10.1534/g3.113.005819)
Supplement: Supporting Information [file supp_3_7_1105__index.html]

Sequence-Based Mapping of the Polyploid Wheat Genome — Supporting Information 

# Sequence-Based Mapping of the Polyploid Wheat Genome

## Supporting Information for Saintenac *et al.*, 2013

**Files in this Data Supplement:**

- Supporting Information - Figures S1-S2, Files S1-S2, and Tables S1-S3 (PDF, 331 KB)
- Figure S1 - Data analysis workflow (PDF, 86 KB)
- Figure S2 - Comparison of marker positions between the *de novo* map and the map developed using bin-mapping approach (PDF, 178 KB)
- Table S1 - Distribution of PA and SNP Variation Across the Wheat Genome (PDF, 67 KB)
- Table S2 - Number of PstI tags Used for the Development of *de novo* Genetic Map (PDF, 65 KB)
- Table S3 - Distribution of PstI Tags Across the 3B chromosome (PDF, 60 KB)
- File S1 - Sequences of barcoded PstI adaptors (.xls, 52 KB)
- File S2 - List of PstI tags showing similarity to the wheat chromosome 3A assemblies (.txt, 151 KB)
