# Supplementary material for: Sequence-Based Mapping of the Polyploid Wheat Genome
Source: G3 (Bethesda). 2013 Jul 1;3(7):1105–14. doi: 10.1534/g3.113.005819 (PMC3704239; doi:10.1534/g3.113.005819)
Supplement: Supporting Information [file supp_g3.113.005819_005819SI.pdf]

G3·Genes | Genomes | Genetics

Sequence-based mapping of the polyploid wheat genome

Cyrille Saintenac[1], Dayou Zhang, Shichen Wang, Eduard Akhunov[2]

Department of Plant Pathology, Kansas State University, Manhattan, KS 66502, USA
[1] Present address: INRA-UBP, UMR1095, Genetics Diversity and Ecophysiology of Cereals, 5 chemin de Beaulieu, 63039 Clermont-Ferrand, France

[2]Corresponding author: Department of Plant Pathology, Kansas State University, Manhattan, KS 66502, USA. E-mail: eakhunov@ksu.edu

DOI: 10.1534/g3.113.005819

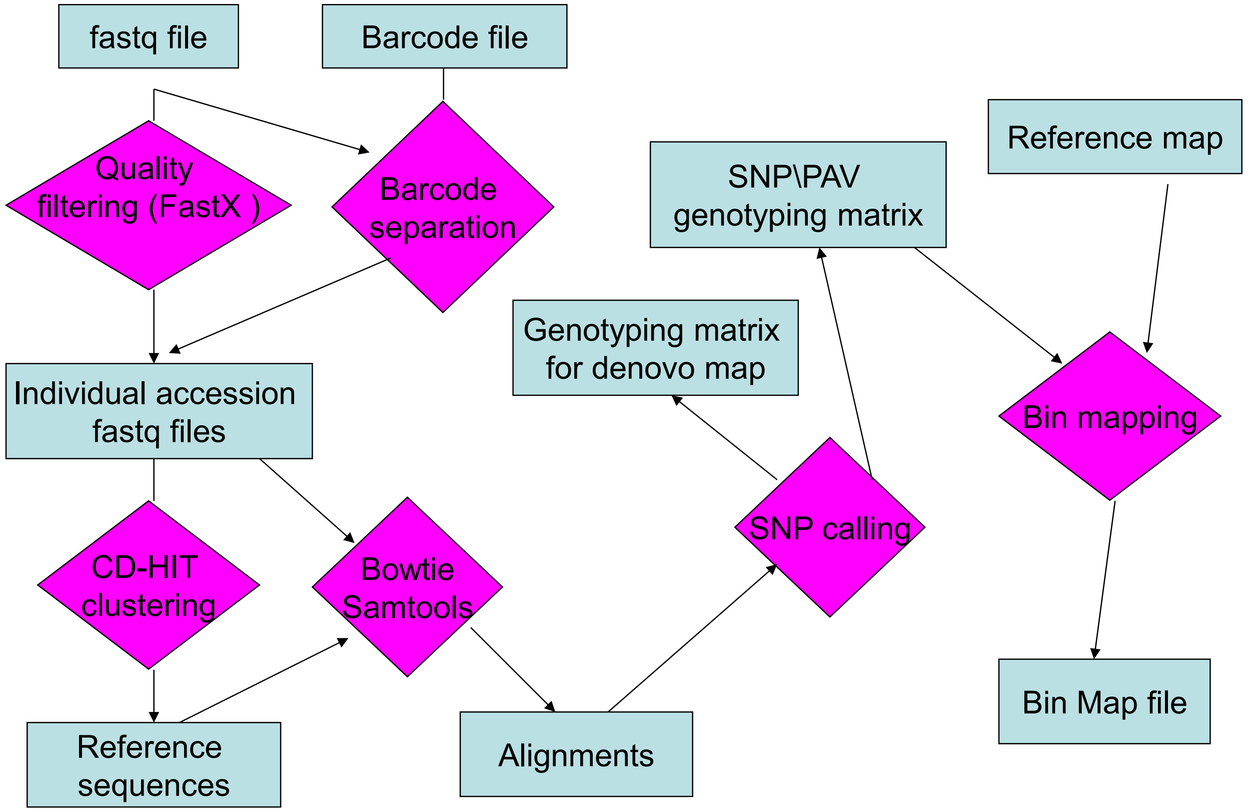

Figure S1 Data analysis workflow.

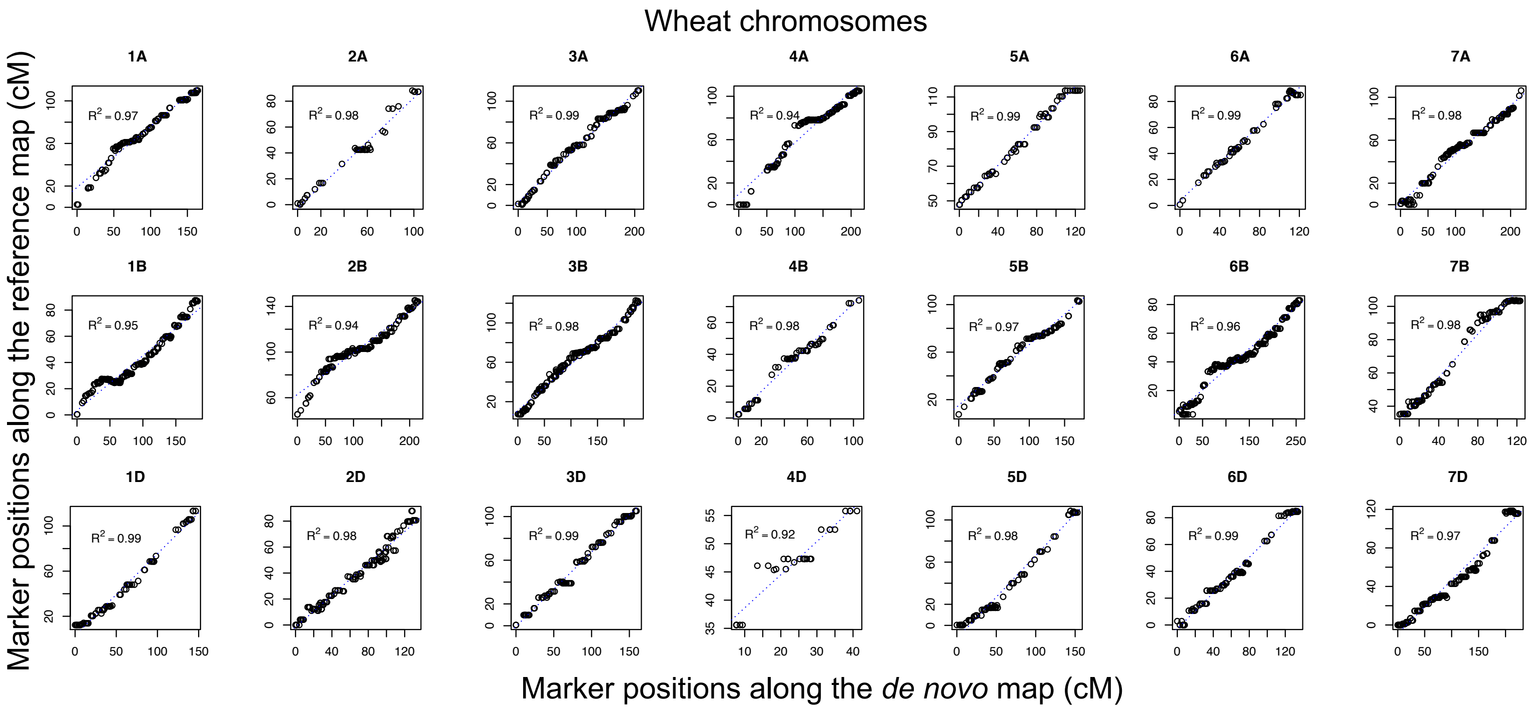

Figure S2 Comparison of marker positions between the *de novo* map and the map developed using bin-mapping approach.

File S1

Sequences of barcoded PstI adaptors

File S2

List of PstI tags showing similarity to the wheat chromosome 3A assemblies.

Files S1 and S2 are available for download at http://www.g3journal.org/lookup/suppl/doi:10.1534/g3.113.005819/-/DC1.

Table S1 Distribution of PA and SNP Variation Across the Wheat Genome

Chromosomes	Presence/Absence (PA) variation				SNP variation				Total
	PstI-MluI	PstI-MseI	PstI-MspI	Total PA	PstI-MluI	PstI-MseI	PstI-MspI	Total SNPs	
1A	478	6,580	7,517	14,575	34	1,505	1,111	2,650	**17,225**
1B	794	10,084	12,264	23,142	79	2,046	1,575	3,700	**26,842**
1D	301	4,438	5,840	10,579	40	718	581	1,339	**11,918**
2A	657	6,881	8,837	16,375	64	1,205	1,003	2,272	**18,647**
2B	920	11,012	14,797	26,729	90	2,122	1,829	4,041	**30,770**
2D	1,750	6,676	11,901	20,327	53	1,233	1,089	2,375	**22,702**
3A	487	5,584	7,977	14,048	54	1,524	1,214	2,792	**16,840**
3B	749	11,686	15,056	27,491	114	2,459	1,996	4,569	**32,060**
3D	534	5,845	7,986	14,365	51	1,408	1,096	2,555	**16,920**
4A	519	8,279	10,094	18,892	44	1,651	1,251	2,946	**21,838**
4B	318	5,122	6,738	12,178	47	1,452	1,119	2,618	**14,796**
4D	213	2,316	3,617	6,146	27	385	307	719	**6,865**
5A	857	7,848	11,214	19,919	75	1,169	991	2,235	**22,154**
5B	842	8,009	11,681	20,532	73	1,514	1,283	2,870	**23,402**
5D	740	4,363	5,558	10,661	44	945	878	1,867	**12,528**
6A	409	5,773	8,488	14,670	48	1,125	977	2,150	**16,820**
6B	543	8,632	10,217	19,392	56	1,648	1,473	3,177	**22,569**
6D	358	4,435	6,404	11,197	39	949	791	1,779	**12,976**
7A	484	8,778	11,088	20,350	94	2,017	1,619	3,730	**24,080**
7B	720	10,663	13,774	25,157	80	2,320	1,806	4,206	**29,363**
7D	383	5,408	7,113	12,904	58	1,434	1,145	2,637	**15,541**
**Total**	**13,056**	**148,412**	**198,161**	**359,629**	**1,264**	**30,829**	**25,134**	**57,227**	**416,856**

Table S2 Number of PstI tags Used for the Development of *de novo* Genetic Map

Chromosomes	Number of SNP mapped de-novo
1A	134
2A	45
3A	118
4A	175
5A	69
6A	62
7A	178
**Total A genome**	**781**
1B	205
2B	200
3B	225
4B	64
5B	117
6B	247
7B	127
**Total B genome**	**1,185**
1D	87
2D	138
3D	103
4D	32
5D	90
6D	105
7D	180
**Total D genome**	**735**

Table S3 Distribution of PstI Tags Across the 3B chromosome (Contigs are ordered physically from the distal arm of the short arm to the distal arm of the long arm)

Contigs	Contig size (bp)	PstI tags	Density of PstI tags per 100 kb	TE content
ctg0011b	1,266,078	121	9.557073103	49.70
ctg0954b	3,109,948	136	4.373063472	63.20
ctg1030b	619,476	3	0.484280263	97.80
ctg1035b	711,534	0	0	89.90
TaaCsp3BFhA_0100L17	268,551	0	0	96.10
ctg0616b	786,544	2	0.254276938	90.70
ctg0382b	1,610,902	6	0.372462136	88.70
ctg0005b	1,715,514	1	0.058291567	92.10
ctg0528b	1,033,236	6	0.580699859	91.20
ctg0464b	2,543,369	4	0.157271713	82.40
ctg0091b	2,776,447	8	0.288138041	89.40
ctg0079b	1,305,738	5	0.382925212	88.00
ctg0661b	465,250	13	2.794196668	74.50
