# Supplementary figures and images for: Sequence-Based Mapping of the Polyploid Wheat Genome
Source: G3 (Bethesda). 2013 Jul 1;3(7):1105–14. doi: 10.1534/g3.113.005819 (PMC3704239; doi:10.1534/g3.113.005819)

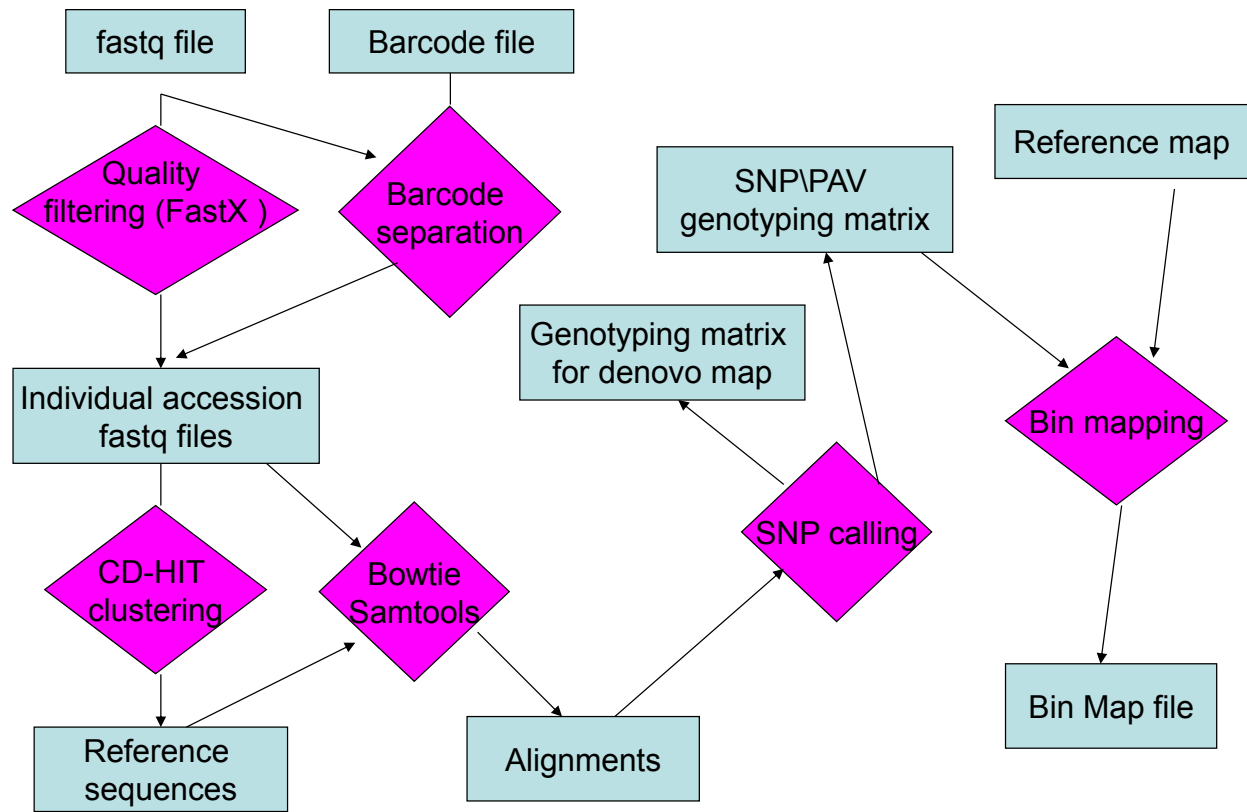

**Figure S1** Data analysis workflow.

Supplement: Supporting Information [file supp_g3.113.005819_FigureS1.pdf]
