# Supplementary material for: Sequence-Based Mapping of the Polyploid Wheat Genome
Source: G3 (Bethesda). 2013 Jul 1;3(7):1105–14. doi: 10.1534/g3.113.005819 (PMC3704239; doi:10.1534/g3.113.005819)
Supplement: Supporting Information [file supp_g3.113.005819_TableS1.pdf]

**Table S1 Distribution of PA and SNP Variation Across the Wheat Genome**

| Chromosomes | Presence/Absence (PA) variation | | | | SNP variation | | | | Total |
|---|---|---|---|---|---|---|---|---|---|
| | PstI-MluI | PstI-MseI | PstI-MspI | Total PA | PstI-MluI | PstI-MseI | PstI-MspI | Total SNPs | |
| 1A | 478 | 6,580 | 7,517 | 14,575 | 34 | 1,505 | 1,111 | 2,650 | **17,225** |
| 1B | 794 | 10,084 | 12,264 | 23,142 | 79 | 2,046 | 1,575 | 3,700 | **26,842** |
| 1D | 301 | 4,438 | 5,840 | 10,579 | 40 | 718 | 581 | 1,339 | **11,918** |
| 2A | 657 | 6,881 | 8,837 | 16,375 | 64 | 1,205 | 1,003 | 2,272 | **18,647** |
| 2B | 920 | 11,012 | 14,797 | 26,729 | 90 | 2,122 | 1,829 | 4,041 | **30,770** |
| 2D | 1,750 | 6,676 | 11,901 | 20,327 | 53 | 1,233 | 1,089 | 2,375 | **22,702** |
| 3A | 487 | 5,584 | 7,977 | 14,048 | 54 | 1,524 | 1,214 | 2,792 | **16,840** |
| 3B | 749 | 11,686 | 15,056 | 27,491 | 114 | 2,459 | 1,996 | 4,569 | **32,060** |
| 3D | 534 | 5,845 | 7,986 | 14,365 | 51 | 1,408 | 1,096 | 2,555 | **16,920** |
| 4A | 519 | 8,279 | 10,094 | 18,892 | 44 | 1,651 | 1,251 | 2,946 | **21,838** |
| 4B | 318 | 5,122 | 6,738 | 12,178 | 47 | 1,452 | 1,119 | 2,618 | **14,796** |
| 4D | 213 | 2,316 | 3,617 | 6,146 | 27 | 385 | 307 | 719 | **6,865** |
| 5A | 857 | 7,848 | 11,214 | 19,919 | 75 | 1,169 | 991 | 2,235 | **22,154** |
| 5B | 842 | 8,009 | 11,681 | 20,532 | 73 | 1,514 | 1,283 | 2,870 | **23,402** |
| 5D | 740 | 4,363 | 5,558 | 10,661 | 44 | 945 | 878 | 1,867 | **12,528** |
| 6A | 409 | 5,773 | 8,488 | 14,670 | 48 | 1,125 | 977 | 2,150 | **16,820** |
| 6B | 543 | 8,632 | 10,217 | 19,392 | 56 | 1,648 | 1,473 | 3,177 | **22,569** |
| 6D | 358 | 4,435 | 6,404 | 11,197 | 39 | 949 | 791 | 1,779 | **12,976** |
| 7A | 484 | 8,778 | 11,088 | 20,350 | 94 | 2,017 | 1,619 | 3,730 | **24,080** |
| 7B | 720 | 10,663 | 13,774 | 25,157 | 80 | 2,320 | 1,806 | 4,206 | **29,363** |
| 7D | 383 | 5,408 | 7,113 | 12,904 | 58 | 1,434 | 1,145 | 2,637 | **15,541** |
| **Total** | **13,056** | **148,412** | **198,161** | **359,629** | **1,264** | **30,829** | **25,134** | **57,227** | **416,856** |
