# Supplementary material for: Sequence-Based Mapping of the Polyploid Wheat Genome
Source: G3 (Bethesda). 2013 Jul 1;3(7):1105–14. doi: 10.1534/g3.113.005819 (PMC3704239; doi:10.1534/g3.113.005819)
Supplement: Supporting Information [file supp_g3.113.005819_TableS2.pdf]

Table S2 Number of PstI tags Used for the Development of *de novo* Genetic Map

Chromosomes	Number of SNP mapped de-novo
1A	134
2A	45
3A	118
4A	175
5A	69
6A	62
7A	178
**Total A genome**	**781**
1B	205
2B	200
3B	225
4B	64
5B	117
6B	247
7B	127
**Total B genome**	**1,185**
1D	87
2D	138
3D	103
4D	32
5D	90
6D	105
7D	180
**Total D genome**	**735**
