# Supplementary material for: Sequence-Based Mapping of the Polyploid Wheat Genome
Source: G3 (Bethesda). 2013 Jul 1;3(7):1105–14. doi: 10.1534/g3.113.005819 (PMC3704239; doi:10.1534/g3.113.005819)
Supplement: Supporting Information [file supp_g3.113.005819_TableS3.pdf]

Table S3 Distribution of PstI Tags Across the 3B chromosome (Contigs are ordered physically from the distal arm of the short arm to the distal arm of the long arm)

Contigs	Contig size (bp)	PstI tags	Density of PstI tags per 100 kb	TE content
ctg0011b	1,266,078	121	9.557073103	49.70
ctg0954b	3,109,948	136	4.373063472	63.20
ctg1030b	619,476	3	0.484280263	97.80
ctg1035b	711,534	0	0	89.90
TaaCsp3BFhA_0100L17	268,551	0	0	96.10
ctg0616b	786,544	2	0.254276938	90.70
ctg0382b	1,610,902	6	0.372462136	88.70
ctg0005b	1,715,514	1	0.058291567	92.10
ctg0528b	1,033,236	6	0.580699859	91.20
ctg0464b	2,543,369	4	0.157271713	82.40
ctg0091b	2,776,447	8	0.288138041	89.40
ctg0079b	1,305,738	5	0.382925212	88.00
ctg0661b	465,250	13	2.794196668	74.50
